# Animal cognition and the evolution of human language: why we cannot focus solely on communication

**DOI:** 10.1098/rstb.2019.0046

**Published:** 2019-11-18

**Authors:** W. Tecumseh Fitch

**Affiliations:** Department of Cognitive Biology, University of Vienna, Wien, Austria

**Keywords:** animal cognition, animal communication, language evolution

## Abstract

Studies of animal communication are often assumed to provide the ‘royal road’ to understanding the evolution of human language. After all, language is the pre-eminent system of human communication: doesn't it make sense to search for its precursors in animal communication systems? From this viewpoint, if some characteristic feature of human language is lacking in systems of animal communication, it represents a crucial gap in evolution, and evidence for an evolutionary discontinuity. Here I argue that we should reverse this logic: because a defining feature of human language is its ability to flexibly represent and recombine concepts, precursors for many important components of language should be sought in animal *cognition* rather than animal communication. Animal communication systems typically only permit expression of a small subset of the concepts that can be represented and manipulated by that species. Thus, if a particular concept is not expressed in a species' communication system this is not evidence that it lacks that concept. I conclude that if we focus exclusively on communicative signals, we sell the comparative analysis of language evolution short. Therefore, animal cognition provides a crucial (and often neglected) source of evidence regarding the biology and evolution of human language.

This article is part of the theme issue ‘What can animal communication teach us about human language?’

## 1. Introduction

I have not, to my knowledge, spoken the word ‘octopus’ today or indeed in the past week, but no one would therefore conclude that I lack the concept OCTOPUS (here I follow the philosopher's convention, when necessary, of denoting conceptual representations in capital letters). Indeed, I have spent many hours observing these creatures and read books about them but, like most of my mental concepts, OCTOPUS goes unexpressed in my speech most of the time. This is not only true of concepts captured by single words (like ‘octopus’, ‘chartreuse’, ‘quasar’ or ‘exponent’) but for more complex cognitive constructs that I possess (like how to walk from the Jardin de Luxembourg to the Place Stravinsky in Paris, via Notre Dame) but have never spoken at all. Humans possess many concepts, within individual minds, that go unexpressed via their language output for long periods of time (and some may never be expressed verbally). However, my assumption in what follows is that pretty much any human concept *could* be expressed in language, with perhaps hours or days of effort, and with varying degrees of accuracy, difficulty and concision. This capability to express any concept goes far beyond what any other species can do.

In what follows, I will take the basic observation that most concepts go unexpressed as axiomatic and argue that the same is true regarding animal communication, only more so (using ‘animal’ as shorthand for ‘non-human animal’ hereafter). For at least in principle, I might, under some circumstances, exclaim ‘Octopus!’ (e.g. when seeing one unexpectedly) or tell you the way to the Place Stravinsky (if you asked me), providing evidence that I indeed possess these concepts. By contrast, it is the nature of all known animal communication systems that they allow their bearers to express only a small *subset* of the concepts they can remember, represent and manipulate productively (cf. [1]). For example, honeybees have excellent colour vision and can remember the colours of the flowers they visit, but the honeybee dance ‘language’ allows a forager to communicate only the spatial location of the flower and has no provision for expressing colour information. I will provide evidence for this below and review similar evidence for other species, including non-human primates. I conclude that animal communication systems appear to be intrinsically limited to a smallish set of fitness-relevant messages that relate to such factors as food, danger, aggression, appeasement or personal prowess. But a substantial literature in animal cognition reveals that they *know* much more than this, even if they have no way of *saying it* [2].

The core argument is that, just as a person's utterances reveal only a subset of what they know, animal communication signals express an intrinsically limited subset of that species' conceptual storehouse. The argument that most thoughts are not expressed is by no means new: it follows Jackendoff's (2002) model of linguistic semantics closely and is also consonant with Chomsky's model [3,4]. Both Hurford [2] and Bickerton [5] have explored its implications for language evolution at book length [2,5], as have I more briefly [1]. My aim here is simply to argue this crucial point sharply and concisely, for although these ideas should not be controversial, they are rejected by some prominent philosophers, and even when accepted, their implications are ignored in many recent discussions of language evolution (e.g. [6,7]).

The central implication of my thesis is that the field of animal cognition has a very important role to play in our understanding of human language evolution because the fact that animals *have* concepts (whether expressible via signalling or not) erases a potentially gaping evolutionary chasm that would exist if they did not. Apparent discontinuities between humans and animal cognition that ‘pose a severe challenge for evolutionary explanation’ ([6], p.3), may in fact be based on discontinuities between language and other species' communication systems. This elision between two different things—cognition and communication—is at best misleading and often pernicious. The study of animal communication is indeed important for comparative analysis of language evolution, most obviously relevant for factors involved in externalization, such as vocal learning, speech perception and gestural communication. But to get the full comparative picture, we need to embrace animal cognition as a central and in some cases *the* central source of information relevant to the biology and evolution of language (and human cognition more generally).

## 2. Words ≠ concepts

Before discussing animals, it is important to first clarify some basic issues about the nature of human concepts, and to at least dip our toes into the philosophical quagmire surrounding the term ‘concept’ (for a concise introduction see [8]). My take on concepts in this essay will be essentially that of mainstream cognitive (neuro)science today, where a concept is simply ‘a nonlinguistic psychological representation of a class of entities in the world’ (Murphy [9], p. 335).

More specifically, my perspective is mentalistic and representationalist. I assume that concepts are mind-internal entities—‘representations’—that often, but not necessarily, correspond to some entities ‘out there’ in the world. It is physicalist: conceptual representations ultimately consist of neural activity in brains (they have no platonic existence, independent of minds). Finally, it is pluralistic, meaning that it allows for different types of concepts, some best captured by definitions, others by prototypes and still others as abilities to discriminate or act. Although much ink has been shed regarding the virtues and flaws of these different interpretations, both in cognitive science [9] and philosophy [10–13], precisely where one stands on these philosophical issues will have little relevance to my comparative argument here.

**Figure 1.**
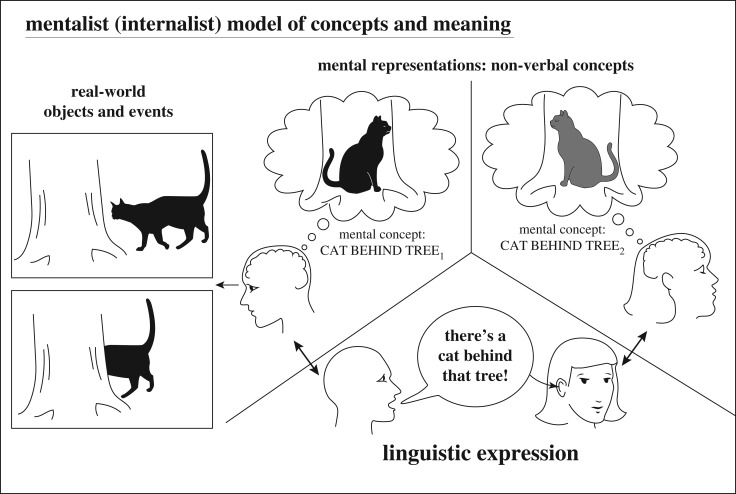
Mentalist model of concepts and meaning: contemporary cognitive scientists argue that words and sentences connection to their referents is indirect, and that reference requires the intervention of a (private) mental concept. Thus, an organism can have a concept (illustrated by the thought bubbles) independently of any words, sentences or other signals that express this concept. Referential links between real-world objects or events and non-verbal mental concepts (representations) can exist even if an organism has no means in its communication system to express those concepts.

However, one central issue, illustrated in figure 1, cannot be ignored, concerning a long-running philosophical debate between ‘mentalists’ (virtually all modern cognitive scientists) and ‘referentialist’ philosophers like Quine or Putnam [12,13]. Referentialists posit a direct referential linkage between utterances and their real-world referents. The referentialist doctrine was dominant in behaviourist psychology of language, which privileged observable behaviours (such as speaking words and pointing) over invisible mental constructs. But it has fallen by the wayside in modern cognitive science—at least regarding human language [3,14]. The alternative mentalist perspective (also termed the ‘internalist’ or ‘conceptualist’ perspective, [3,4]) holds that words do not refer directly to things in the world, but rather express our (mind-internal) concepts. To paraphrase Strawson ‘words don't refer, *people* refer’ [15]. The concepts we express linguistically may correspond to real entities in the world, but in many cases (e.g. ‘Sherlock Holmes’, ‘the unicorn in my dream’), they do not.

The modern mentalist perspective in cognitive science sees acts of referring (e.g. by speaking) as being indirect. That is, reference involves two separable phenomena (figure 1): first a mental representation of an entity is recognized, recalled or otherwise activated, and second some utterance is produced which may, if successful, elicit a similar though not identical mental representation in the listener. For example, observing a cat walk behind a tree, I may form a mental representation of CAT BEHIND TREE. This complex concept is the first step in reference: a correspondence between real-world events (e.g. visual patterns interpreted as cats and trees) and the resulting mental representation. Generating this particular non-verbal concept is accomplished by the visual system, is private, and (I argue below) essentially the same type of cognitive processing that occurs when a dog sees a cat go behind a tree (who perhaps indicates this knowledge by straining at its leash).

The second stage of reference—externalization—is the one with a public, perceivable component: under some circumstance, I may choose to say ‘there's a cat behind that tree’ or perhaps ‘hinter dem Baum ist eine Katze’ (in German). This second step in referring links my mental representation to some signal in English, German, American sign language, etc. Crucially, my mental representation is the same for either sentence (the very idea of translation—that different sentences in different languages can refer to the very same concept—assumes some language-independent conceptual world). Again the link between the concept CAT BEHIND TREE and either of these sentences is initially an internal matter, within the speaker's mind, and dependent on their personal conceptual and linguistic competences. However, if finally I utter one of these sentences, the utterance enters the public sphere and may cause an appropriately equipped listener to form their own mental representation CAT BEHIND TREE (probably different in detail from mine). Linguistic communication—concept sharing—has occurred.

This indirect model may sound overly complicated or obscure. We have a strong intuition that words themselves ‘mean things’ and sentences ‘refer’, regardless of whether anyone reads or understands them. This intuition about direct reference is hard to shake and still taken quite seriously by some philosophers. This may be because the intuition is biologically grounded, stemming from a ‘referential drive’ to interpret words as meaningful, part of the species-typical ‘instinct to learn’ that underpins child language acquisition [1]. For the child inferring word meanings, the simple notion that words mean things provides a useful shortcut to get the semantic system up and running. This intuition persists into adulthood, leading to superstitious beliefs (the magical powers of names or ritual chants). Despite providing a concise shorthand for denoting the more circuitous process detailed above, the referentialist intuition is completely inadequate as a full description of linguistic meaning [3]. Freeing ourselves from the shackles of this prescientific intuition is the first step to insightful scientific analysis.

Embracing this indirect, two-step nature of reference, I can now state my argument more clearly: the first stage of reference—building representations that tie sensory input to conceptual representations—is built upon a chassis of cognitive processes (sensory processing, recognition, categorization, combination and inference) that has fundamental shared components between humans and other animals. These components long predated language. The second stage of ‘externalization’—the capacity to form signals representing these non-verbal concepts—represents a crucial difference in humans and was one of the key innovations in human language evolution [16]. As Jackendoff puts it ‘phonology and syntax… evolved to *express* meaning, which has a far longer evolutionary pedigree’ ([3], p. 428).

It was once common to take a link between concepts and language as definitional, such that a ‘true’ concept must be linked to a word [17,18], but this traditional notion seems unsustainable in the face of infant research, where infants can clearly represent and reason about things they have no words for [19–22].

## 3. Do animals have concepts?

The considerations above lead most cognitive scientists to assume that the meanings of words and sentences are to be cashed out in non-linguistic mental representations: ‘concepts’ hereafter. However, the cognitive revolution remains incomplete: while few today deny the existence of internal mental representations (concepts) in humans, many remain suspicious when attributing them to animals. Animal cognition researchers are typically required to reject all possible associative explanations, regardless of their complexity, before attributing mental representations to animals [23] and the discipline spends considerable energy and ingenuity refuting so-called killjoy associative explanations [10,24]. Fortunately, the field has matured to the point where, for many phenomena, there can be little doubt that mental representations exist in animals, and can be recalled, manipulated and themselves represented [25–27].

Concepts should be, in some sense, general and flexible, and might initially be equated with mental ‘categories’. It is uncontested that birds and mammals learn and recall categories [28,29], but some have claimed that animal categories are little more than reflexes, reactively elicited in sensory cortices by sensory inputs and lacking the flexibility and generality of human concepts [18,30]. However, current data demonstrate that many species form cross-modal associations, showing that their categories are flexibly multi-modal [31–33]. Animals can summon categorical representations in the absence of relevant triggering stimuli, for instance seeking hidden food items at particular times, or re-hiding food items a potential thief saw them hide, in the absence of that thief [34]. They can compute abstract relationships like ‘same’ and ‘different’, for example, correctly choosing novel ‘same’ pairs when presented with two matched objects, and vice versa when given unmatched pairs [35,36]. Many species can compute transitive inferences: knowing that if A > B and B > C, then A must be greater than C as well [37–39]. These data fulfil the philosophers' desideratum that (animal) concepts should be more than unimodal, reflexive, stimulus-driven dispositions to react appropriately: they have an abstract categorical and relational structure.

A sceptical philosopher might still object that however impressive these cognitive abilities are, they do not ‘really’ constitute concepts. Concepts require not just categorization (first-order representations), but a second-order representation of that knowledge: knowing *that* (or doubting that, or being surprised that) some perceptual object belongs to the category. Animal concepts are limited, philosophers like Davidson argue, to first-order representations [40]. The most telling evidence against this ‘first-order’ view comes from studies on ‘metacognition’, where animals exhibit an understanding of their own conceptual representations (beliefs about beliefs). If uncertain about their own knowledge, they will choose a ‘don't know’ response, for lesser reward, rather than guessing [41]. Most research in this experimental paradigm been done on rhesus macaques but related work documents metacognition in dolphins, rats and pigeons (cf. [42]). Such experiments involve a response to some discrimination task, yielding a food reward, but an additional response is allowed for uncertain cases, often glossed as ‘I don't know’. The animal can choose the ‘don't know’ option when uncertain, receiving a smaller food reward than they would receive for a correct answer, but no punishment. Typically, in situations of high uncertainty (e.g. stimuli ambiguous from a human perspective), animals in these experiments choose the ‘uncertain’ button.

Although some critics have suggested that animals in such experiments simply form a new perceptual category (e.g. ‘unfamiliar’) and pushing the button for this, this possibility can be ruled out in most of the primate experiments (for the refutation of this and other ‘killjoy’ hypotheses, cf. [43]). Recent experiments are most compelling. Monkeys are first trained on one set of experimental stimuli, for example, based on colour discrimination, to learn the ‘don't know’ option. If this response was really tied to perceptual cues (e.g. colour) about the training stimuli, there should be no carry-over of this third option to novel stimulus sets. Instead, monkeys immediately transfer their appropriate use of the third option to novel situations (e.g. area discrimination) or even from past (retrospective) judgements to future (prospective) judgements [44]. This strongly suggests that the animals truly doubt their knowledge (represent their own uncertainty) and can transfer a response based on this meta-knowledge to novel situations. These and other data have convinced even previous sceptics that animals possess representations about representations, and therefore ‘concepts’ in this more demanding Davidsonian sense [45]. Of course, human metacognition is more sophisticated, involving thoughts about thoughts about thoughts… But that fact provides no empirical grounds to deny basic second-order metacognition to other animals. Given these modern data, denials that animals possess basic non-verbal concepts seem misinformed and anti-scientific (e.g. [30]).

I hasten to add that my claim here is *not* that animal concepts are of the same complexity or flexibility as those of humans. That would be absurdly anthropomorphic and would ignore the fact that language, as a multi-component system [16], also includes recursive compositional machinery that allows us to flexibly combine basic concepts into complex, hierarchically structured thoughts. This compositionality is a key component of linguistically structured thought, independent of externalization. Indeed, Chomsky terms it the ‘Basic Property’ of language and argues that it was selected in the human lineage precisely for its value in structuring internal thought, rather than externalizing these thoughts via speech [4,46]. There is at present little evidence of complex compositionality in animal communication or cognition (beyond things like transitivity, discussed below) [47]. But crucially, if we want to understand the evolution of this component, the appropriate starting point is animal *conceptual* abilities, and cannot be limited to the signals animal produce.

I now turn to the empirical data supporting my main contention that animals possess more concepts than their communication systems allow them to express. For reasons of space and concision, this is a very selective review—the data are so abundant that a full treatment requires an entire book (for this I recommend [2,29]). I will thus focus on a few examples from clever species, like primates and dolphins, plus honeybees, because these are well documented in easily accessible publications.

## 4. Animal signals ≠ animal concepts

To empirically demonstrate that a species can conceptualize more than they can express requires both an understanding of their communication system and independent data concerning their cognition. A nice example to start with is the honeybee *Apis mellifera*, in which communication and cognition are well-studied. The honeybee communication system allows a forager who has discovered flowers, upon returning to the hive, to inform other foragers of their location [48,49]. In the darkness of the hive, the bee performs a stereotyped (and apparently innate) ‘waggle dance’ whose direction, relative to gravity, signals the azimuth direction of the flowers (relative to the sun). The duration of the waggle portion correlates with the distance to the flowers, and by combining these cues, the dance provides a remarkably accurate indication of the location of these flowers. This system is also remarkable in ‘referring’ to an entity not currently present or visible to the communicators (thus sharing the property of ‘displacement’ with human language; [50]). Finally, the system is flexible, because a honeybee can ‘refer’ to the location of other objects than flowers when necessary, for instance, water or a new nest-site (I put ‘refer’ in quotes to avoid philosophical debate—I simply mean that a honeybee's dance reliably allows naive honeybees to locate the object in question).

Despite this impressive communication system, detailed studies of honeybee cognition reveal even more impressive cognitive abilities (reviewed in [51]). For example honeybees have excellent colour vision and can remember the colour of rewarding versus unrewarding nectar sources over days [52,53]. Nonetheless, their dance ‘language’ has no way to communicate colour information. Even more impressive, a honeybee can judge whether two stimuli are the same or different in colour or pattern [54] and generalize this behaviour to novel modalities (trained on colour, she immediately transfers the same/different decision to patterns or vice versa). Again, however, the honeybee dance language lacks signals for ‘same’ or ‘different’. Thus, even an insect whose brain occupies 1 mm^3^ and contains less than a million neurons has cognitive abilities that significantly outstrip its ability to communicate them.

Turning now to a large-brained species, the bottlenose dolphin *Tursiops truncatus* is another species for which we have solid data about both cognition and communication. Dolphins have sophisticated cognitive abilities rivalling those of non-human primates [31]. They rapidly learn a ‘delayed match-to-sample’ task and generalize across hundreds of novel sounds [55]. Dolphins can remember lists of items (spatial locations, visual objects or sounds), correctly indicating whether a probe stimulus was or was not in the list, and show a classic recency effect, like humans [31]. Dolphins show cross-modal integration, matching visually and acoustically perceived (via echolocation) object shapes, and show mirror self-recognition, inspecting themselves in a mirror when marked in an otherwise invisible location (and not doing so when sham-marked). Dolphins readily learn to interpret human signals, whether gestural (e.g. pointing) or auditory [56] and can understand novel combinations of signals (‘sentences’ made up of multiple gestures or sounds) on the first try, based on a simple order-based grammar (e.g. responding correctly to ‘take the hoop to the ball’ versus ‘take the ball to the hoop’). Dolphins can understand the abstract command ‘create’ indicating ‘do something novel’ by performing some new action or ‘repeat’ to perform the act again (thus requiring the dolphin to keep track of what it itself had done). All of these data indicate that dolphins have a flexible, productive capacity to learn, can self-monitor and can retain and manipulate novel concepts across multiple modalities.

However, turning to bottlenose dolphins' well-studied communication system, we get a very different picture. Early studies indicated a quite complex vocal communication system, and the ability of dolphins to learn human words suggested that they might have a ‘language’ of their own [57]. These suggestions led to careful experiments attempting to understand dolphin communication via observation and playback experiments that, on the contrary, suggested an ordinary mammalian repertoire of vocal signals [58], with the exception that dolphins are vocal learners and readily learn to mimic both conspecific and human-generated sounds [31,59,60]. Vocal learning is put to use in a ‘signature whistle’ system: dolphins emit an individual-specific whistle pattern (for example, when captured) that can be imitated by other dolphins, leading to exchanges and reuniting of separated animals [61]. Young dolphins initially acquire their whistles, by imitation [62,63]. Although this is an interesting system, with a capacity to signal individuals (reminiscent of ‘names’), it appears to be the most productive aspect of their vocal system.

The evidence against greater expressive ability comes from experiments where two dolphins are allowed to communicate vocally while solving a joint task [64–66]. Individual dolphins readily learn to push on a right or left paddle depending on a visual signal. With more training, two dolphins who can see each other can learn a social version: a signal perceived by one dolphin must be responded to by the other dolphin first, and only afterwards by the second, to provide a food reward to both. The crucial experimental condition involves blocking visual contact between the two individuals. If dolphins possessed a flexible language-like communication system, it should be a simple matter to signal ‘push the left one’ and succeed. Although initial experiments suggested this [64], more careful follow-up studies showed that these initial successes did not reflect anything language-like. When the roles were reversed (so that the former responder had to become the signaller), the pair totally failed. Furthermore, when the contingency between signal and response was changed, the dolphins had to be retrained from scratch and were not able to simply switch vocal signals to indicate the other action. The researchers concluded that the initial success was a result of trial-and-error learning where incidental sounds made by one animal, or vocal sounds produced whether or not the other animal was present, were used to solve the task [58,65]. Bastian, who led this research project concisely concluded ‘No evidence was found to support the supposition that the social signalling of dolphins is capable of the transfer of arbitrary environmental information’ (p. iii, [65]). Summarizing, dolphins have very sophisticated cognitive and learning abilities, revealing complex internal concepts, but their capacity to communicate those concepts via their species-typical signals is quite limited.

## 5. Concepts and communication in primates

My final examples come from two non-human primate species—vervet monkeys and chimpanzees—but similar examples could be provided for many other well-studied primates.

Vervets *Chlorocebus pygerythrus* (previously *Cercopithecus aethiops*) are small common African monkeys, possessing a suite of different alarm calls that are typically emitted in the presence of different predators [67,68]. The vervet monkey alarm call system is frequently cited as a potential precursor to language [10,69]. However, the three different alarm calls produced to leopards, eagles and snakes in no way exhaust the concepts that vervets can represent. In addition to ‘standard’ primate concepts like individuality and dominance [70], vervets maintain complex spatial representations of their environment [71] and can mentally track the locations of hidden group members [72]. They can socially learn how to access food and rapidly absorb new social preferences about what to eat based on colour [73]. None of this cognitive sophistication is in any way detectable in their vocal communication system.

Turning finally to our nearest living relatives, the chimpanzees and bonobos (*Pan troglodytes* and *Pan paniscus*), there is abundant evidence that chimpanzees have highly developed cognitive abilities and can represent basic concepts like colour and shape, as well as abstract concepts including sameness, location, and sequence [27,74,75]. Chimpanzees also have social representations including individual identity, dominance and relationships (e.g. ‘child of’) and are capable of transitive inference [76]. With extensive training, very abstract concepts like number are within their cognitive reach [77,78]. They show at least the beginnings of theory of mind, in that they can represent what competitors do or do not see [79]. Their tool-using abilities are sophisticated and incorporate future planning [80]. When trained intensively with human communication systems, they can understand multi-word sentences and indicate an impressive variety of objects and events [81,82] and exhibit flexible cross-modal transfer of information without further training [83]. In general then, chimpanzees exhibit some of the most sophisticated cognitive abilities known among animals—unsurprising given their close biological relationship to humans.

By contrast, chimpanzee vocal communication is comparable to that seen in many other primates or mammals, with a small repertoire of 30-odd innate vocalizations [84] including food calls that differ for different food quality [85,86], screams and threats, and complex display calls like pant-hoots [87]. Chimpanzees are not known to have predator-specific alarm calls like vervets. Their gestural communication system is considerably richer, and perhaps more intentionally informative than their vocal communication [88–90]. But both their vocal and gestural communication skills pale in comparison to their rich and sophisticated cognitive abilities. Cognitive studies demonstrate beyond a reasonable doubt that chimpanzees possess many concepts that their species-typical communication systems cannot express (nor indeed do the utterances of ‘language trained’ chimpanzees come close to expressing the complexity of concepts like number, transitivity or tool use [82,91]). Thus, chimpanzees clearly possess and manipulate concepts that they are unable to communicate. Even the most exhaustive analysis of chimpanzee communication would vastly underestimate the complexity of their non-verbal conceptual world.

It is crucial not to conflate these communicative limitations with the false but frequently repeated claim that primates (or animals more generally) have no voluntary control over their vocalizations. A sizeable body of data clearly demonstrates that they do (cf. [92]). For example, in the wild, many species (including chickens and monkeys) exhibit ‘audience effects,’ producing vocalizations only when appropriate listeners are around [70,93,94], and chimpanzee screams and alarm vocalizations are clearly modulated by the presence and composition of the audience [95,96]. Several bird species produce ‘false’ alarm calls when no predator is present, frightening away competitors and then taking remaining food [97,98]. In an operant setting in the laboratory, numerous studies have demonstrated voluntary production (or inhibition) of vocalizations on command [97,98] in chimpanzees, other primates [99,100] and various other mammals (e.g. cats and dogs, [101]). Thus, despite a common misconception, animal vocalizations are not reflexive actions, performed inevitably upon the appearance of some external stimulus; but this fact does not imply that their vocalizations provide exhaustive access to their conceptual world.

## 6. Conclusion: discontinuities in signalling do not indicate cognitive discontinuities

I end by clarifying the key implication of this essay: when considering the evolution of human cognition, we will be fundamentally misled if we attribute to animals only those concepts they can communicate. Externalization of concepts is just one component of language, and another is to help structure our private internal thought [4]. Thus, we cannot accurately limit our estimation of what humans *know* to what they *say*. The same is true of animals, only more so. The flexibility of human language means that we can use it to represent virtually anything we can think (perhaps with considerable effort, in the case of visual, musical or highly abstract concepts). The same flexibility and expressivity is simply not present in animal communication systems. This limitation, rather than any fundamental non-existence of animal concepts, was surpassed by humans during language evolution. Thus, our (linguistic) ability to refer, not our basic ability to conceptually represent, must be explained if we hope to understand the neural and ultimately genetic basis of human language.

This is not to deny that externalized language gives humans a huge conceptual advantage over other species. We acquire many concepts via language that we have no direct access by personal experience, vastly enlarging our potential store of knowledge (some readers may never have personally seen an octopus, but most will nonetheless have some concept OCTOPUS). Blind people, thanks to language, have surprisingly rich conceptions of colour terms [102], and many abstract or scientific terms such as ‘electron’ or ‘truth’ have no sensory manifestations at all. My argument is not that animals have precisely the same concepts as humans (that would be absurd, because even individual humans do not share precisely the ‘same’ concepts, figure 1). My argument concerns the neural and cognitive machinery underlying the formation of mental representations, along with many of the cognitive processes that allow concepts to be formed based on sensory experience and combined at a basic level. These capabilities are shared across species and were therefore present before language evolved and provided the precursors of more complex human concepts.

In many circumstances, the study of animal communication can provide crucial insights into what animals know and remains an important part of comparative investigation of language evolution. But accepting the fundamental fact that animals know much more than they can express implies that the evolution of human language built upon a pre-existing conceptual apparatus much richer than that observable in animal communicative capabilities. It is therefore critical that future scholarly explorations of human language evolution take results from animal cognition research as crucial data for understanding the evolutionary path to human language. Even more crucial is a dedicated research programme to explore in detail animals' abilities to combine concepts. To the extent that they can do so in a flexible, hierarchical manner [103,104], I think we can see the germs of the recursive symbolic system that underlies human linguistic concepts.

## Data accessibility

This article has no additional data.
